# Impact of Internet Use on Mental Health among Elderly Individuals: A Difference-in-Differences Study Based on 2016–2018 CFPS Data

**DOI:** 10.3390/ijerph19010101

**Published:** 2021-12-23

**Authors:** Huan Zhang, Hongyang Wang, Huiyu Yan, Xiaoyu Wang

**Affiliations:** School of Social Development and Public Policy, Beijing Normal University, Beijing 100875, China; zhanghuan@bnu.edu.cn (H.Z.); wanghongyang0115@163.com (H.W.); 18866852982@163.com (H.Y.)

**Keywords:** elderly, Internet, life satisfaction, depression level, difference-in-differences method

## Abstract

The number of elderly Internet users has increased significantly in the past few years. However, the impact of Internet use on mental health remains unclear. In this study, we performed a difference-in-differences analysis using data from the 2016 and 2018 waves of the China Family Panel Studies (CFPS) to evaluate the impact of Internet usage on mental health among elderly individuals. A total of 5031 validated respondents were included to explore the relationship between Internet use and reduced levels of depression as well as improved life satisfaction among elderly individuals. The results showed that Internet use significantly reduced depression levels. Unexpectedly, Internet use was not found to improve life satisfaction. Moreover, discontinuing Internet use was not significantly associated with improvements in depression or life satisfaction. More research is needed to fully elucidate the relationship between Internet use and depression levels, as well as life satisfaction among elderly individuals.

## 1. Introduction

In the information age, the rapid spread of Internet technologies has strongly impacted all aspects of people’s lives, and new technologies such as online social networking, travel reservations, and mobile payments have brought great convenience to the daily lives of elderly individuals. According to “The 47th China Statistical Report on Internet Development”, issued by the China Internet Network Information Center, the proportion of “Elderly Internet users” reached approximately 260 million, as of December 2020, accounting for 18.4% of the total population. This percentage will continue to increase. Due to the use of the Internet, seniors can complete many tasks without leaving the home. As elderly individuals continually age, and their mobility becomes restricted, the Internet could be a convenient way to meet the diverse needs of seniors, and could be especially beneficial to seniors who prefer to remain in one place [1,2,3]. Considering the availability of home-based community care services, elderly people could use the Internet to establish connections with the outside world, integrate into society and increase their social participation. Furthermore, elderly individuals can also benefit from the diagnosis, treatment, and monitoring of diseases through telemedicine [4]. However, elderly individuals may also encounter numerous embarrassing and difficult situations because of the digital divide [5]. They develop negative emotions such as feeling disconnected from society and feeling useless as they grow older [6]. Occasionally, seniors may experience undesirable events such as Internet rumours and Internet scams, which diminish the experience of older Internet users [7]. Due to the rapid development of Internet technology, the subjective feelings and emotional reflections of older people need to be focused on.

Previous studies have shown that several factors affect the use of the Internet by elderly individuals. The first factor is the characteristics of elderly individuals themselves. Elderly men are more inclined to use the Internet than elderly women [8]. With declining vision and hearing, it is more difficult for elderly individuals to use the Internet [9], thus hindering their enthusiasm for the Internet and online participation in their daily lives [10]. Additionally, the more educated the elderly are, the more increasingly likely they are to use the Internet [11]. Seniors who have no physical pain prefer using the Internet than those with chronic illnesses or disabilities [5]. The second important factor is income level: higher-income older individuals are freer to use the Internet [12]. The third factor is the living area. In terms of place of residence, elderly individuals living in main urban areas appear to be more prone to using the Internet [13]. The last factor is social support: elderly people who have more friends prefer to use the Internet. The state’s financial support and the development of communication technology infrastructure also affect the use of the Internet by the elderly [14].

Research has shown that diverse factors affect the mental health of elderly individuals. It is commonly believed that age, sex, and physical health status may affect people’s mental health [15,16,17]. Additionally, the majority of seniors choose to live at home rather than in a nursing home; therefore, the community environment plays an increasingly important role in improving the mental health of elderly individuals [18,19].

Previous studies have identified a wide array of indicators between Internet usage and the mental health of elderly individuals. Several researchers believe that the Internet has a beneficial effect on social relationships, through providing convenient and effective means for communication among elderly individuals [20]. For example, Internet use could improve life satisfaction [21,22,23,24], enhance well-being [25,26,27], and reduce loneliness [28], as well as the depressive symptoms of elderly individuals [29,30,31,32,33]. In contrast, other studies found that Internet use may play negative roles in elderly people’s lives. These studies insist that increased time spent online erodes elderly people’s social interactions. Internet use leads to a reduction in social participation, a narrowing of social circles, and a weakening of the sense of community belonging and networks of friends [34,35]. For example, the digital divide brings distress and tension to elderly individuals [36,37]. Additionally, another view is that the impact of Internet use on mental health among elderly individuals cannot be generalized, because the research is inconsistent in terms of life satisfaction, depression, and loneliness. Shapira et al. [21] studied elderly people who received training in Internet use in day centres or nursing homes, and found significant improvements in the sense of mastery, depressive symptoms, and loneliness among the interventionists, but no significant results in terms of quality of life. Choi et al. [38] searched through the literature published in peer-reviewed journals and found no effects of Internet use noted in reducing depression in elderly individuals. Forsman et al. [39] conducted a systematic review of articles published in international databases, and found that 42 of 101 measures related to psychological perceptions were not significant, including quality of life, depression, functional independence, and loneliness. These results are interpreted with caution and explored with specific concern to the different age stages of Internet use and Internet content among elderly individuals.

In this paper, we investigate why there are inconsistent findings on the effects of Internet use on the mental health of elderly individuals, and what the influencing factors are. According to current studies, most results are based on data from a certain year, but ignore the causal and time effects. This study attempts to advance the literature by exploring the causal effect of Internet usage on the mental health of elderly individuals. Our research focuses on two main questions. We ask, does Internet use improve mental health among elderly individuals compared with those who do not use the Internet? Moreover, does withdrawing from Internet use diminish the mental health of elderly people compared with elderly people who use the Internet consistently? We used the difference-in-differences method to verify the impacts of Internet use or disuse on the mental health of elderly people over time. The paper is divided as follows: Section 2 presents the data, the main variables, and models of the influencing factors of Internet usage; Section 3 presents the verified stability of the research results; Section 4 and Section 5 provide a brief discussion and concluding remarks.

## 2. Materials and Methods

### 2.1. Samples and Data Sources

The data were derived from the China Family Panel Studies (CFPS) (Data access link: http://www.isss.pku.edu.cn/cfps/ (accessed on 4 May 2018)), a social survey sponsored by the Institute of Social Science Survey (ISSS) of Peking University. The baseline survey was officially launched in 2010, and all family members and their future blood/adopted children, as genetic members of the CFPS, are also permanently tracked. The survey aims to track and collect data at three levels—individuals, families, and communities—to reflect the changes in China’s economy, society, population, development of education, and individual health. The CFPS sample covers 25 provinces, municipalities, and autonomous regions, with a target sample size of 16,000 households, and includes all household members in the sample. This survey has been ethically reviewed, and provides real and reliable data for academic research and national and social policy decisions. In 2016, it began to include a survey of Internet usage.

In this study, we chose whether elderly individuals used the Internet as the independent variable based on the theory of Use and Disuse. Lamarck first proposed this theory to explain the evolutionary principles of human organs. It involved two principles: the first principle considered that the behaviour and habits of organisms cause changes in the organism and its parts, and the second principle considered that evolution is influenced by life, territory, and environment [40]. According to the theory of Use and Disuse, elderly individuals maintain the best control of their lives by actively interacting with their environment. The concept of “evolution in usage” implies that elderly people actively participate and integrate into society, use the Internet to enrich their daily communication, strengthen connections with children and friends and live a better life in their later years.

The dependent variables were selected concerning the meaning of mental health. The World Federation of Mental Health defines mental health into four categories: first, regulating the body, intelligence, and mood; second, adapting to the environment and maintaining humility in interpersonal relationships; third, having a sense of happiness; and fourth, giving full attention to one’s ability to work, and living an efficient life. As reported by the guidelines for assessing the mental health of elderly individuals in China, mental health is usually measured by the following dimensions: cognitive efficacy, emotional experience, self-awareness, interpersonal interaction, and adaptability. A positive mental health state is reflected as life satisfaction, whereas a negative mental health state is manifested as loneliness and depression. Combining the items from the CFPS database, we measured the mental health of elderly individuals in terms of two dimensions: depressive status and life satisfaction. We applied the difference-in-differences model and data from the 2016 and 2018 waves of the China Family Panel Studies (CFPS), compared the changes of the two groups of elderly people (elderly individuals who began to use the Internet and elderly individuals who discontinued internet use) at two time points, and analysed the pure effect of Internet use on the mental health of elderly individuals. The following hypotheses were proposed for this study:

**Hypothesis** **1** **(H1).** *Elderly individuals who did not use the Internet in 2016 but used it in 2018 were less depressed than those who did not use it in both 2016 and 2018.*

**Hypothesis** **2** **(H2).** *Elderly individuals who did not use the Internet in 2016 but used it in 2018 had higher life satisfaction than those who did not use it in both 2016 and 2018.*

**Hypothesis** **3** **(H3).** *Elderly individuals who used the Internet in 2016 but no longer used it in 2018 had higher levels of depression than elderly individuals who used it in both 2016 and 2018.*

**Hypothesis** **4** **(H4).** *Elderly individuals who used the Internet in 2016 but no longer used the Internet in 2018 had lower life satisfaction than elderly individuals who used the Internet in both 2016 and 2018.*

The research subjects of this article were elderly individuals 60 years and older [41]. To track changes in the mental health of elderly individuals, dropouts and new entrants were excluded. The overall comparison of the basic conditions of elderly individuals in the sample in 2016 and 2018 is shown in Table 1.

Statistics from the 2016 and 2018 samples show that elderly individuals were between the ages of 68 and 70, with a male to female ratio close to 1:1. In terms of health status, the smallest percentage of seniors rated themselves as very healthy, while the percentage of seniors who rated themselves as unhealthy was over 30%. Compared to 2016, the mean value of respondents’ life satisfaction in 2018 increased by 0.39, the mean depression level score increased by 0.1, and the percentage of elderly individuals who used the Internet increased by 6%.

### 2.2. Model Construction

In this paper, the endogeneity problem caused by the problem of self-selection bias and omitted variables is addressed by using the difference-in-differences (DiD) method. The method was first proposed by Heckman [42] for assessing public policy effects. As a quasi-experimental analysis method, the approach is often used to track the resulting changes caused by the change in the policy effect over time. It is based on the counterfactual framework to explore the changes of the dependent variables in the two states of policy that occur, or not. The difference between the core observed variables before and after implementation is calculated by dividing different economic individuals who are subjected to the policy shock, and those who are not, into a treatment group and a control group. The pure effect of the policy is obtained by eliminating the fixed effect and the influence of common time trends among different economic individuals. Compared with other experimental designs, the DiD method can solve the problem of missing variables that do not change with time and avoid the effects of external factors and selection deviation. Given these advantages, the method is commonly used for the quantitative evaluation of the effects of the implementation of a particular public policy or project, and is widely used in the field of econometrics, as well as in the field of sociology.

In this study, elderly individuals were divided into groups A and B. In group A, the experimental group was those who used the Internet in 2018 but did not use the Internet in 2016: they were named the Internet usage access group. The control group was those who did not use the Internet in either year: they were named the Internet unused group. The experimental group in group B is elderly individuals who used the Internet in 2016, but quit using it in 2018, they were named the Internet usage exit group. The control group was seniors who were using the Internet in both 2016 and 2018: they were named the Internet usage persistence group. On the one hand, the effect of Internet use on the mental health of elderly individuals was analysed by comparing the change in individuals between 2018 (using the Internet) and 2016 (not using the Internet). On the other hand, the effect of stopping Internet use on the mental health status of elderly individuals was analysed by comparing the change in individuals between 2018 (stopped using the Internet) and 2016 (used the Internet). Other factors that may have an impact on individuals’ mental health status, including age, sex, education, and health status, were also controlled to further mitigate the bias of the results caused by omitted variables. In addition, the DiD estimation was needed to ensure that elderly individuals surveyed at both time points were in the same cohort, so the IDs in the questionnaire were used to exclude elderly individuals who were new to the CFPS survey, and those who dropped out in 2018. The DiD model was set as follows:

$$Y_{i}{}_{t}=\beta_{0}+\beta_{1}\mathrm{Treatment}+\beta_{2}\mathrm{Time}+\beta_{3}\mathrm{Treatment}\cdot \mathrm{Time}+\varepsilon_{\mathrm{it}}$$

where *β*_0_ indicates the control group, which is a constant term; *β*_1_ indicates the gap of the elderly people’s mental health itself; Treatment indicates whether or not to use the Internet, and takes the value of 1 for using the Internet and 0 for not using the Internet; *β*_2_ indicates the time effect; Time indicates the time of elderly individuals using the Internet, and takes the value of 0 before using the Internet and 1 after using the Internet; *β*_3_ represents the pure effect of change in the mental health of elderly individuals after removing the time effect and the disparity in elderly individuals themselves; and ɛ_it_ indicates the error term.

In the study, demographic variables such as sex and age, Internet use, depression level, and life satisfaction of elderly individuals were first described to obtain a basic picture of the data. Second, the DiD method was used to determine the causal relationship between the use of the Internet and changes in the mental health of elderly individuals from 2016 to 2018. Furthermore, the pure effect of Internet use on the mental health of elderly individuals was evaluated based on the difference in the change.

### 2.3. Variables and Operationalization

#### 2.3.1. Use of the Internet

Both the 2016 questionnaire and the 2018 questionnaire in CFPS asked the following questions: “Do you use mobile devices, such as cell phones, tablets, to surf the Internet?” and “Do you use a computer to surf the Internet?” Respondents answered “yes” or “no”, and those who used a computer, the cell phone, or a tablet to surf the Internet were defined as using the Internet and assigned a value of 1; those who did not use the Internet were assigned a value of 0.

#### 2.3.2. Mental Health Status

The dependent variable in this study was the mental health of elderly individuals, and two scores, life satisfaction of elderly individuals and depression level of elderly individuals, were used as measures for the pure effect of Internet use on the mental health of elderly individuals. The subjective attitude module of the CFPS covered the measurement of life satisfaction. The measure of life satisfaction was divided into five levels, representing the degree of satisfaction with one’s life, and was rated on a scale of 1 to 5. Depression level was measured using the CES-D scale short version of the CFPS questionnaire, which included 8 questions (6 measures of negative mood and 2 measures of positive mood) with 4 response options, namely, 0 = none, 1 = occasionally, 2 = often, and 3 = most of the time. Respondents’ depression scores were calculated by summing together the scores from each question; the items “I feel happy” and “I live happily” were taken as the measure of positive emotion, and the negative values were taken and added together when calculating the scores. Higher scores indicated higher levels of depression.

#### 2.3.3. Control Variables

Since the age, sex, education, and health status of elderly individuals are endogenous to a considerable degree, affecting both their mental health and their Internet use, they were used as control variables. The variables were defined as follows: the sex of the respondent was taken as 1 for males and 0 for females. Age referred to the difference between the year the respondent was born and the year when the respondent was interviewed. Education referred to the highest education level of the respondent at the time of graduation, including the lower level of education such as illiterate/semiliterate, elementary/junior high school, moderate level of education such as high school/junior high school/technical school/vocational high school, and higher education level such as college, bachelor’s degree, master’s degree, doctorate: in order, the values were taken as 1–8 points. Health status referred to the respondents’ perception of their health, which was divided into 5 levels, with the lowest value being 1, meaning “very healthy”, and the highest value being 5, meaning “unhealthy”.

## 3. Results

### 3.1. Descriptive Analysis

Excluding dropouts and new entrants from the follow-up survey, a total of 5031 individuals were included in the sample for this study. First, descriptive analyses were performed on the percentage of Internet use, life satisfaction, and depression level of the sample in 2016 and 2018. Subsequently, differences in control and outcome variables were compared based on reported sample sizes for the Internet usage access group, the Internet unused group, the Internet usage exit group, and the Internet usage persistence group.

#### 3.1.1. Sample Status

Table 2 reports the basic information on Internet use among elderly individuals in the sample in 2016 and 2018. In terms of age, the largest proportion of seniors aged 60–69 used the Internet, while none of the seniors aged 90 or older used the Internet. In terms of sex, the percentage of Internet use among elderly individuals was higher among men than women. In terms of education, the largest proportion of elderly people with a medium level of education used the Internet. In terms of health status, relatively healthy seniors tended to use the Internet more. The characteristics of seniors using the Internet in 2018 were similar to those in 2016, but the number of Internet users increased.

In terms of life satisfaction, elderly people aged 90 and above had the highest average scores of life satisfaction in 2016. In 2018, elderly individuals aged 80–89 had the highest life satisfaction. Older men had lower levels of satisfaction than women. Again, in terms of the self-rated health of elderly individuals, when elderly individuals were in extremely good health, they had the highest life satisfaction scores; when elderly individuals were in extremely bad health, they had the lowest life satisfaction scores in comparison.

In terms of depression level, the younger the age group in 2016, the higher the depression levels of the sample group. The lowest depression level was of the sample group aged 90 years and above. In 2018, elderly individuals aged 80–89 had the highest depression levels, and the sample group with the lowest depression levels was the same as that in 2016. Older men had lower levels of depression than women. When analysed in terms of education level, older Chinese individuals with high education levels had lower depression scores than those with low education levels. From the perspective of the health level analysis, the mean value of depression levels was the highest for unhealthy elderly people, and the mean value of very healthy elderly people was the lowest for depression.

#### 3.1.2. Comparison of Chi-Square Values of Different Subgroups

The study showed that 297 elderly people were in the Internet usage access group, and 4396 elderly people were in the Internet unused group. Additionally, 59 belonged to the Internet usage exit group, and 279 elderly people were in the Internet usage persistence group. Combining control variables and outcome variables, the samples of the four different groups were compared for significance with differences. The results are shown in Table 3.

Table 3 compares the chi-square values of the four groups of samples. The chi-squared value of depression level was 150.609, the chi-squared value of life satisfaction was 26.975, the degree of freedom was 3, and the asymptotic significance was 0.00 < 0.01. Therefore, the distribution of the depression level and life satisfaction of elderly individuals in the sample was different in the four different subgroups.

### 3.2. DiD Estimation

#### 3.2.1. Model Estimation

To explore the impact of Internet use on the mental health of elderly individuals, we constructed four DiD models. After controlling for the age, sex, education, and health status of the sample, the DiD estimates were conducted for elderly individuals in the sample. The results are shown in Table 4.

We can see that Internet use in Panel A had a significant effect on the level of depression among elderly individuals when compared with the Internet unused group. The coefficient of the interaction term of the DiD model was −0.4374, with a significance value of 10%, which indicates that Internet use had a negative leading effect on the depression level of the sample population. Elderly individuals who used the Internet reduced their depression level by 0.4374 points, compared with those who did not use the Internet. Compared with the Internet unused group in Group A, the effect of Internet use on the life satisfaction of elderly individuals had a coefficient of −0.0359 for the interaction term of the DiD model, but it is not significant. This indicates that Internet use does not affect the life satisfaction of elderly individuals.

Table 5 shows the effect of continued Internet use on life satisfaction and the depression levels of elderly individuals, compared with discontinued Internet use.

As seen in Table 5, the coefficient of the interaction term of the DiD model for the effect of Internet usage exit group on the level of depression in elderly individuals, compared with the Internet usage persistence group, was 0.2717, which was not significant. The coefficient of the interaction term of the DiD model for life satisfaction was −0.0210, which was also not significant, indicating that discontinuing Internet use did not affect the depression level or life satisfaction of the elderly individuals.

We then examined whether the results on life satisfaction were confounded by the control variables. The four control variables were brought into the model for validation, and were significant when controlling sex and health, but no longer significant when age and education were added. This suggests that the correlation between Internet use and life satisfaction was driven to some extent by sex and health. We analysed the model by adding seven questions (the corresponding items in the questionnaire are QQ1011-QQ1017) that measured the activities of daily living of the elderly individuals from the CFPS database, and found that the results were still not significant.

#### 3.2.2. Placebo Effect Test

In the DiD analysis, it is important to consider the impact of other random factors on mental health after Internet use by the elderly. The placebo effect test plays an important role in the model estimation, which could improve the robustness of the estimation results. The core idea is to fictionalize the processing team or estimate the time for fictitious policies. If the interaction term of DiD still changes significantly under the two treatments, this indicates that the conclusion drawn may have been affected by other factors, and some unconsidered bias. Thus, if a fictitious treatment group that had not been continuing to use the Internet, or a fictitious time when older adults began using the Internet still had a significant effect on their mental health, this would suggest that random factors were at work. If the results are not statistically significant, then the study results are robust.

Since only the Internet use of elderly individuals in the access group had a significant effect on the level of depression in the DiD estimates in this paper, only a placebo effect on the elderly people’s Internet usage group was tested. We tested this by fictionalizing the amount of time elderly individuals spent using the Internet. In this study, the time cut-off point for whether elderly individuals used the Internet was 2018, and we advanced the time by one year to 2017. If there was still a significant effect of Internet use on elderly people’s levels of depression, then this would indicate that the reduction in elderly people’s depression levels was not solely due to Internet use, and therefore the results could not demonstrate the pure effect of the Internet on elderly individuals. Conversely, it indicated that elderly people’s level of depression was not influenced by other factors, and that the reduction was due to Internet use.

Table 6 reports the results of the placebo effect test. As shown in Panel A, the interaction term of the DiD model was not significant in regression with the level of depression of elderly individuals, and the placebo effect test passed, indicating that the decrease in depression levels in elderly individuals was due to the use of the Internet.

## 4. Discussion

The main objective of this study was to use the DiD method to explore the effects of Internet use on the mental health of elderly Chinese people over time. Using CFPS data from 2016 and 2018, our study showed that elderly individuals who are younger, male, more educated, and in better health, are more likely to use the Internet. Using the DiD model, our study found that Internet use had a significant alleviating effect on the depression levels of elderly individuals, which is consistent with previous research [29,30,31,32,33,43], and confirms the theoretical view of “Use and Disuse”. Unexpectedly, there was no significant relationship between Internet use and life satisfaction. This corresponds to previous studies in which the impact of Internet use on the mental health of elderly individuals cannot be generalized [21,38,39]. Moreover, a recent study from the Netherlands also found that ICT use in older age groups 65 years and older appears to be less likely to have an impact on their psychological adjustment (loneliness, life satisfaction, depression) [44]. Collectively, these results suggest that the impact of Internet use on the mental health of elderly people needs to be explored in the context of different mental health dimensions.

Additionally, the impact of Internet use on the mental health of elderly individuals is subject to a complex series of factors. The first is urban–rural difference. For example, the Internet affected the mental health and physical health of urban elderly people, and only affected physical health for rural elderly people [45]. The second is income difference: Internet use is effective in reducing mental health problems among low-income older people, while the effect is not significant for older people with higher income levels [46]. The third is the influence of Internet access preferences. Typically, elderly people spend more time online for recreational and leisure activities [47]. If they only use the Internet to search for information, the psychological situation of older people does not significantly change [19]. The fourth factor is the influence of the properties of the Internet itself: poor web usability and barriers to Internet use among older adults can lead to a lack of knowledge and confidence, fear of science and technology, and a distrust of social media or websites, reducing the overall experience [48]. If the functional aspects of the website design are very elaborate, elderly people will access healthier information and self-manage psychological states such as “low mood”, “stress” and “anxiety” [49]. The fifth is external stimulus. External motivators, such as social connections, could increase motivation and self-efficacy to use the internet among elderly people [50]. It is evident from the above study that the relationship aspect of the impact of Internet use and the mental health of elderly individuals needs to be continuously and deeply explored. Nearly all elderly people have the mentality of not giving in to old age, and aim to prove their worth throughout their later years. According to the theory of Use and Disuse proposed by Lamarck, the use of the Internet can enhance the social participation of elderly individuals and help them rediscover their value while enabling them to communicate with the outside world and access resources and daily needs. Therefore, researchers may need to consider the impact that these factors might have on the mental health of elderly individuals in terms of their Internet use. In addition, it is also important to further explore the direction of the relationship among the effects of these influential factors.

This study enriches the literature on the effects of Internet use on the subjective well-being of elderly individuals. Additionally, the study enables academia to notice different findings on the impact of Internet use on the mental health of elderly individuals. Currently, the trend of an ageing society in China is becoming increasingly serious, and despite the tradition of preferring community-based home care for elderly individuals, there is an increasing trend of residential separation between children and parents. Based on the concept of age friendliness and the initial intention of caring for elderly individuals, this research on the impact of Internet use on the psychological health of elderly individuals is in line with the expectations of society. Almost every elderly person has a mindset of defying old age, hoping to connect with the outside world through media means and spending their old age in a fulfilling and pleasant way. Therefore, this research also has important practical significance.

Admittedly, there are some limitations to the study. First, the mental health of elderly individuals is influenced by multiple factors. Although we used four control variables (age, sex, education, and health), it is difficult to exclude possible confounding by other factors because of the high number of missing values and the fact that many macrolevel socioeconomic indicators are not addressed. Furthermore, elderly individuals in the sample span a large age range, and the lower and higher age groups have different levels of Internet acceptance and awareness, but we did not discuss them specifically in our analysis. Second, Internet use is a long-term behaviour. The CFPS only began to investigate Internet use in 2016, so the impact of Internet use on the mental health of elderly individuals was not as pronounced as expected. Third, we explored the impact of the Internet on the mental health of elderly individuals only in terms of whether they use the Internet, but not in terms of specific functions. These limitations may have confounded the study results to some extent. Attention needs to be paid to these issues in future research, by selecting a database with a large sample size and a long duration to further validate the relationship between Internet use and mental health in elderly individuals.

## 5. Conclusions

Internet use significantly reduces depression levels in elderly individuals. However, Internet use appears not to be associated with improved life satisfaction. Moreover, discontinuing Internet use had no significant effect on either depression levels or life satisfaction in elderly individuals. This study adds new empirical evidence to the research on the relationship between Internet use and the mental health of elderly individuals. Studies focusing on the effects of withdrawal from Internet use versus continued Internet use behaviour on the mental health of elderly individuals remain limited. Therefore, further research is needed to explore the relationship between persistent internet usage and the mental health of elderly individuals. Additionally, the results of studies on the effects of Internet use on the mental health of elderly individuals are still debated extensively. Our study encourages scholars to pay attention to inconsistent findings and analyses that lead to different results.

## Figures and Tables

**Table 1:** Characters of study subjects.

	2016		2018	
	Mean or %	SD	Mean or %	SD
Age	69.36	7.54	68.23	6.36
Female	51.90		49.74	
Education				
Low	76.03		70.56	
Middle	21.79		27.09	
High	2.18		2.35	
Health status				
Very healthy	15.24		17.08	
Relatively healthy	29.8		35.82	
Generally healthy	23.29		17.10	
Unhealthy	31.67		30.00	
Life satisfaction	3.85	1.06	4.24	0.93
Depression level	−0.26	4.67	−0.15	4.54
Use of Internet	0.06		0.12	
N	5031		5031	

**Table 2:** Results of the descriptive analysis on variables.

	2016							2018						
	% Of Internet Use	Life Satisfaction			Depression Level			% Of Internet Use	Life Satisfaction			Depression Level		
		Mean	SD	Mean Diff	Mean	SD	Mean Diff		Mean	SD	Mean Diff	Mean	SD	Mean Diff
Age														
60–69	5.49	3.80	1.07	0.222 ***	−0.54	4.51	0.005	8.49	4.21	0.94	0.108 ***	−0.16	4.44	0.053
70–79	1.11	4.00	0.99	−0.184 ***	−0.44	4.68	−0.130	2.62	4.30	0.89	−0.078 ***	−0.13	4.79	−0.020
80–89	0.12	4.15	0.90	−0.289 ***	−1.10	4.56	0.581 *	0.34	4.39	0.86	−0.152 ***	0.03	4.73	−0.175
90 and above	0.00	4.22	0.67	−0.355	−1.89	6.07	1.352	0.00	4.19	0.66	0.061	−0.75	5.35	0.613
Gender														
Male	4.27	3.83	1.03	0.072 **	−1.17	4.34	1.297 ***	7.10	4.22	0.93	0.064 **	−0.79	4.35	1.344 ***
Female	2.44	3.90	1.07	−0.072 **	0.13	4.70	−1.297 ***	4.35	4.28	0.91	−0.064 **	0.55	4.71	−1.344 ***
Education														
Low	1.37	3.89	1.07	−0.084 **	−0.12	4.68	1.402 ***	3.52	4.29	0.93	−0.151 ***	0.30	4.74	−1.615 ***
Medium	4.17	3.79	0.99	0.100 ***	−1.60	4.02	1.402 ***	6.66	4.13	0.89	0.157 ***	−1.23	3.90	1.442 ***
High	1.17	3.96	0.92	−0.091	−2.66	3.60	2.171 ***	1.27	4.22	0.85	0.025	−2.31	3.41	2.223 ***
Health status														
Very healthy	0.76	4.25	0.91	−0.462 ***	−2.46	3.61	1.651 ***	1.49	4.51	0.79	−0.319 ***	−2.04	3.81	2.281 ***
Relatively healthy	3.30	3.91	0.97	−0.065 **	−1.66	3.83	1.651 ***	5.57	4.27	0.86	−0.032	−1.16	3.86	1.605 ***
Generally healthy	1.75	3.79	1.03	0.100 ***	−0.65	4.10	0.145	1.77	4.16	0.92	0.104 ***	−0.27	4.24	0.160
Unhealthy	0.91	3.63	1.17	0.319 ***	2.14	5.06	−3.667 ***	2.62	4.13	1.02	0.177 ***	2.22	4.96	−3.372 ***
Sample size	5031							5031						

Note: *** *p* < 0.01, ** *p* < 0.05, * *p* < 0.1.

**Table 3:** Comparison of the cardinal values of the four sample groups.

Test Statistic a, b		
	Depression Level	Life Satisfaction
χ^2^	150.609	26.975
df	3	3
Asymptotic Significance	0.00	0.00

Note: a: Kruskal–Wallis test; b: Grouping Variable.

**Table 4:** Results of the DiD estimation (Group A).

Group A	Depression Level	Life Satisfaction
Internet Usage Access Group (ref. Internet Unused Group)	−0.4374 *	−0.0359
	(−1.69)	(−0.61)
Age	−0.7143	1.1410 ***
	(−1.34)	(9.40)
Gender	−0.7039 ***	−0.0866 ***
	(−7.65)	(−4.14)
Health status	1.2463 ***	−0.1433
	(34.37)	(−17.38)
Education	−0.5624	−0.0319
	(−12.27)	(−3.06)
R-squared	0.1467	0.0760
N	4693	4693

Note: *** *p* < 0.01, * *p* < 0.1.

**Table 5:** Results of the DiD estimation (group B).

Group B	Depression Level	Life Satisfaction
Internet usage exit group (ref. Internet usage persistence group)	0.2717	−0.0210
	(0.98)	(−0.16)
Age	1.1390	1.0835 **
	(0.62)	(2.016)
Gender	−0.2407	−0.0987
	(−0.90)	(−1.36)
Health status	1.0687 ***	−0.2137 ***
	(7.94)	(−5.85)
Education	−0.3786 ***	0.0183
	(−3.53)	(0.63)
R-squared	0.1071	0.0859
N	338	338

Note: *** *p* < 0.01, ** *p* < 0.05.

**Table 6:** Results of Placebo effect test.

Group A	Depression Level
Internet Usage Access Group (ref. Internet Unused Group) DID_be	−0.1865
	(−0.73)
Age	−0.2146
	(−0.41)
Gender	−0.7107 ***
	(−7.71)
Health status	1.2450 ***
	(34.29)
Education	−0.5628
	(−12.27)
R-squared	0.144
N	4693

Note: *** *p* < 0.01.

## Data Availability

The data were released to the researchers without access to any personal data. Data access link: http://www.isss.pku.edu.cn/cfps/ (accessed on 4 May 2018).
